# 31 Regional Anesthesia for Reducing Postoperative Opioid Use in Split Thickness Skin Grafting

**DOI:** 10.1093/jbcr/irac012.034

**Published:** 2022-03-23

**Authors:** Robert P Duggan, Austin T Mefford, Alen Palackic, Adebukola Owolabi, Ludwik K Branski

**Affiliations:** University of Texas Medical Branch, Galveston, Texas; University of Texas Medical Branch, Galveston, Texas; University of Texas Medical Branch, Galveston, Texas; University of Texas Medical Branch, Galveston, Texas; University of Texas Medical Branch, Galveston, Texas

## Abstract

**Introduction:**

Split thickness skin grafting is ubiquitous in the management of acute burns and burn reconstruction. Patients describe the resulting partial-thickness donor site wound as one of the most painful aspects of burn care. Managing donor site pain is challenging and frequently involves potent opioid regimens. Rapid reepithelization of the donor site makes long-acting local and regional anesthesia an attractive option for reducing opioid use. This study aims to determine the efficacy of graft donor site regional anesthesia at reducing postoperative opioid consumption in burn patients.

**Methods:**

A retrospective review of burn patients undergoing split-thickness skin grafting at our institution was performed. Patient demographics, burn mechanism, and percent burned total body surface area were collected. The type of regional anesthesia, when it was performed, and the anesthetic agents used were also determined. Milligram morphine equivalents (MME) were calculated for three 24-hour periods postoperatively to quantify opioid usage. The total MME in 72h postoperatively was also determined and used to calculate per day MME requirements. Mean, and peak pain scores in the first 24h postoperatively were collected. Univariate and multivariate analyses were performed to determine the efficacy of regional anesthesia.

**Results:**

Twenty-five patients were identified, 14 who received donor site regional anesthesia and 11 who did not. The two groups did not differ significantly in age, gender, race, BMI, or burn mechanism. The regional anesthesia group had a significantly lower percent burned TBSA (5.3 vs. 21.6, p < 0.001). Still, donor site dimensions did not differ significantly between groups (363 cm2 vs. 411 cm2, p = 0.247). The use of regional anesthesia was associated with significantly lower MME requirements in the first 24h postoperatively (22.5 vs. 84.9, p = 0.023), lower total requirements after 72h (47.3 vs. 147.8, p = 0.016), and lower per day requirements (17.6 vs., 51, p = 0.014). The regional anesthesia group was discharged on average one week sooner (5.1 days vs. 12.4 days, p = 0.031). Multivariate analysis demonstrated the use of regional anesthesia independently predicted decreasing MME requirements in the first 24h after surgery, decreasing MME requirements in total, and decreasing per day MME requirements. No patients experienced anesthesia-related complications.

**Conclusions:**

In a cohort of burn patients undergoing split-thickness skin grafting, the use of regional anesthesia was highly effective at reducing opioid requirements in the immediate postoperative period. We believe regional anesthetic blockades should be considered to provide long-lasting donor site analgesia. More investigation is warranted into ideal anesthetic agents, the maximum donor site dimensions, and the extent of cost savings.

